# Hindlimb unloading-induced reproductive suppression via Downregulation of hypothalamic *Kiss-1* expression in adult male rats

**DOI:** 10.1186/s12958-021-00694-4

**Published:** 2021-03-04

**Authors:** Amira Moustafa

**Affiliations:** Department of Physiology, Faculty of Veterinary Medicine, Zagazig University, Zagazig, 44519 Egypt

**Keywords:** Testosterone, Kisspeptin, Testes, Microgravity, Heat shock protein

## Abstract

**Background:**

Spaceflights-induced microgravity can alter various physiological processes in human’s body including the functional status of the reproductive system. Rodent model of tail-suspension hindlimb unloading is extensively used to stimulate the organs responses to the microgravity condition. This study explores the potential effects of hindlimb unloading on testicular functions and spermatogenesis in adult male rats and the underlying mechanism/s.

**Methods:**

Twenty Sprague-Dawley rats were allotted into two groups: normally loaded group (control; all arms were in touch with the grid floor) and hindlimb unloaded group (HU; only the forearms were in contact with the grid floor).

**Results:**

Following 30 days of exposure, the HU group saw a decline in body weight, testicular and epidydimal weights, and all semen parameters. The circulating concentrations of gonadotropin-releasing hormone (GnRH), follicle stimulating hormone (FSH), luteinizing hormone (LH) and testosterone significantly decreased, while levels of kisspeptin, corticosterone, inhibin, prolactin and estradiol (E2) increased in the HU group. Intratesticular levels of 5α-reductase enzyme and dihydrotestosterone (DHT) were suppressed, while the levels of aromatase and kisspeptin were significantly elevated in the HU group. Hypothalamic kisspeptin (*Kiss1*) mRNA expression levels were downregulated while its receptors (*Kiss1R*) were upregulated in the HU group. On the contrary, the mRNA expression levels of testicular *Kiss1* were upregulated while *Kiss1R* were downregulated. The pituitary mRNA expression levels of *FSHβ* and *LHβ* decreased in the HU group. The levels of the antioxidant enzymes, superoxide dismutase (SOD), catalase (CAT), glutathione peroxidase (GPx) and nitric oxide (NO) concentrations, and total antioxidant capacity (TAC) were elevated while malondialdehyde (MDA) concentrations declined in the testes of HU group. The testes of the HU rats showed positive immunostaining of caspase-3, heat shock protein 70 (HSP70) and Bcl2.

**Conclusions:**

Altogether, these results revealed an inhibitory effect of hindlimb unloading on kisspeptin signaling in the hypothalamic-pituitary-testicular axis with impaired spermatogenesis and steroidogenesis.

## Introduction

Gravity serves as a crucial force that impacts various life functions; altered gravity represents an environmental challenge, which may affect an organism’s physiology. During space flight, astronauts are exposed to many stress factors, such as microgravity and circadian alteration, that affect the physiological function of both human and animal [1, 2]. Reduced gravity provokes a redistribution of blood and fluid from the caudal to the cephalic fraction of the body [3], which alters the expression of several genes if continued for several days [4]. In addition, space flights induce deleterious effects on the musculoskeletal, cardiovascular and immune systems [5, 6]. Due to the restricted opportunities to perform space experiments, the hindlimb unloading model was developed and frequently employed to stimulate the same conditions of microgravity in animals [7] such as the absence of weight, atrophic changes in muscles and bones [8] and disturbances in posture and gait [9].

The hypothalamic–pituitary–gonadal (HPG) axis is vital for the reproductive organ functions, maintenance of gonadal hormone synthesis and gametogenesis. Kisspeptin encoded by the *KiSS-1* gene plays a fundamental role in reproduction via the stimulating HPG axis [10], which is mediated by the hypothalamic gonadotropin releasing hormone (GnRH). GnRH antagonists block the central and peripheral effects of kisspeptin on LH and FSH [11]. Moreover, circulating sex steroids regulate hypothalamic *Kiss-1* expression in rodents and primates, indicating that kisspeptin is engaged in the HPG negative feedback cycle [11, 12].

Due to the increase in long duration space flights, interest in the impact of altered gravity on the reproductive system has grown. Therefore, this study aims to investigate the influence of microgravity on Kisspeptin-HPG axis, its contribution to male fertility disorders and the underlying mechanisms.

## Materials and methods

### Ethical approval

All manipulations and experiments were approved by the Institutional Animal Care and Use Committee of the Faculty of Veterinary Medicine, Zagazig University (ZU-IACUC/2/F/7/2020).

### Experimental design

Eight-week-old male Sprague-Dawley (SD) rats were purchased from the laboratory animal unit at Zagazig University, kept at 22 °C with a 12 h light/ dark cycle and with free access to food and water. After two weeks of adaptation, rats were randomly divided into two groups (10 rats/group): control group and hindlimb unloaded (HU group).

Hindlimb unloading was implemented as described previously [13] with slight modifications. In brief, rats were suspended by the tail with 30° angle in a head-down position permitting only the forelimbs to be in contact with the grid floor, while allowing a free movement to gain access to water and ad libitum. In the control group, the tail was attached to a traction tape but without suspension, allowing both forelimbs and hindlimbs to touch the grid floor but exposed to the same stress by fixing their bodies similarly to the HU group. After the end of the experiment (30 days), all animals fasted overnight, then were anesthetized and killed by exsanguinations.

### Sampling

Blood samples were collected and serum and plasma samples were separated for hormonal assay. For semen evaluation, the caudal part of the epididymis of one testis was immediately utilized. Testes, epididymides, seminal vesicles and prostates were collected for histopathological and immunohistochemical examination. Hypothalamus, pituitary gland and part of testis were immediately harvested on liquid nitrogen and kept at − 80 °C for total RNA extraction.

### Measurement of organs weight and gonadosomatic index

Body weight was recorded weekly throughout the experiment. At the end of the experiment, reproductive organs (testes, epidydimis, seminal vesicle and prostate) were immediately excised and weighed in both the control and HU groups. The gonadosomatic indices were calculated as organ weight/body weight multiplied by 100.

### Semen analysis

The caudal part of the epididymis from one testis was excised and macerated in a petri dish containing 2 ml of a pre-warmed physiological saline solution at 37 °C. The sperm count was performed using hemocytometer and sperm abnormalities were assessed using an eosin-nigrosin stain following a methodology previously indicated [14]. The motility of the spermatozoa was examined directly under a light microscope at a magnification of 400x.

### Estimation of hormones and enzymes

The levels of kisspeptin-1, FSH, LH, and total testosterone were measured using commercially available rat ELISA assay kits (Cusabio Biotech Company, Wuhan, China) according to the manufacturer’s instructions. Intratesticular levels of aromatase, 5α-reductase enzyme and dihydrotestosterone (DHT), and serum levels of GnRH, estradiol (E2), prolactin, inhibin and corticosterone were estimated using rat ELISA assay kits (MyBioSource, San Diego, CA, United States) following the manufacturer’s protocol. The levels of sex hormone binding globulin (SHBG) were assessed in rat plasma using commercially available ELISA kits (Wuhan Fine Biological Technology, Wuhan, Hubei, China) according to the manufacturer’s instructions.

### Measurement of oxidative stress, lipid peroxidation and nitric oxide

Testicular level of superoxide dismutase (SOD) was estimated using rat ELISA assay kit (Cusabio Biotech Company, Wuhan, China). Testicular levels of catalase (CAT), glutathione peroxidase (GPx) and nitric oxide (NO) were measured with rat ELISA assay kits ((MyBioSource, San Diego, CA, United States) following the manufacturer’s protocol. Testicular levels of malondialdehyde (MDA), the lipid peroxidation marker, were measured by commercially available rat ELISA kits (Elabscience, Wuhan, China) according to the manufacturer’s instructions. Testicular and seminal total antioxidant capacity (TAC) were assessed using *Cell Biolabs OxiSelect*™ assay kits (San Diego, Inc., CA, USA), according to the manufacturer’s instructions.

### RNA extraction, cDNA synthesis and real time PCR

Tissues from rat hypothalamus, pituitary and testes were used to isolate RNA using Trizol reagent (ThermoFisher Scientific; Waltham, MA, United States), according to the manufacturer’s instruction. HiSenScript™ RH (−) cDNA synthesis kit (iNtRON Biotechnology Co., South Korea) was used for cDNA synthesis. 1 μg of total RNA was incubated at 45 °C for 60 min, then at 85 °C for 10 min in a Veriti 96 -well thermal cycler (Applied Biosystems, Foster City, CA).

The expression levels of *Kiss1*, *Kiss1R*, *GnRH*, *GnRHR*, *FSHβ*, *LHβ* and *aromatase* mRNA were quantified using a Mx3005P qRT-PCR System (Agilent Stratagene, USA), SYBR® Green PCR Master Mix (Enzynomics*;* Daejeon*,* Korea) and specific primers (Table 1, Eurofins Genomics, Ebersberg, Germany). They were cycled through an initial denaturation of 15 s at 95 °C, followed by 40 cycles of amplification at 95 °C for 20 s and at 55 °C for 30 s, and then the final elongation step at 72 °C for 30 s. Experimental data were normalized by the Gapdh expression value, and the comparative cycle threshold (CT) method was used to calculate the relative expression levels [15]. ΔCT is obtained by subtracting each mean CT value of Gapdh from the corresponding target specific CT (*Kiss1*,*Kiss1R*, *GnRH*, *GnRHR*, *FSHβ*, *LHβ* or *aromatase*) and ΔΔCT is determined by subtracting each ΔCT of the HU group from that of the control group. The fold expression was calculated using the formula 2 − ΔΔCT.

**Table 1:** Primer sequences for gene expression by qRT-PCR

Gene	Forward primer (5′–3′)	Reverse primer (5′–3′)	Accession No	Product size (bp)
***Aromatase***	GCCTGTCGTGGACTTGGT	GGTAAATTCATTGGGCTTGG	NM_017085.2	142
***FSHβ***	AGACCAAACACCCAGAAAG	TCACTATCACACTTGCCACA	NM_001007597.2	140
***LHβ***	CATAGTCTCCTTTCCTGTGGC	CATTGGTTGAGTCCTGGGA	NM_012858.2	91
***GnRH***	GGCTTTCACATCCAAACAGAATG	TGATCCTCCTCCTTGCCCAT	NM_012767.2	181
***GnRHR***	TCAGGACCCACGCAAACTAC	CTGGCTCTGACACCCTGTTT	NM_031038.3	182
***Kiss 1***	TGCTGCTTCTCCTCTGTGTGG	ATTAACGAGTTCCTGGGGTCC	NM_181692.1	110
***Kiss 1R***	CTTTCCTTCTGTGCTGCGTA	CCTGCTGGATGTAGTTGACG	NM_023992.1	102
***GAPDH***	GTGCCAGCCTCGTCTCATAG	CGTTGATGGCAACAATGTCCA	NM_017008.4	122

### Histopathological examinations

Testes, epididymides, seminal vesicles and prostates were fixed with a 10% neutral buffered formalin solution, then dehydrated and embedded in paraffin. Serial paraffin sections (5 μm) were obtained, immersed in xylol, hydrated with a descending alcohol series and finally stained with hematoxylin-eosin (HE). Changes in tissue structures were visualized with a digital camera attached to a photomicroscope (Olympus, Tokyo, Japan).

### Immunological assays

Immunohistochemical analysis of Bcl-2, caspase-3 and HSP70 were conducted in the testes using a UltraVision LP Large Volume Detection system (Thermo Fisher Scientific, Fremont, CA, USA) according to the manufacturer’s protocol. Briefly, paraffin sections of 5 μM thickness were immersed in a 10 mM citrate buffer solution (pH 6.0), heated in an autoclave sterilizer for 10 min, cooled for 30 min, and immersed in 3% aqueous hydrogen peroxide (H2O2) at room temperature for 30 min to block endogenous peroxidase. The sections were then washed in PBS, quenched with blocking buffer (Ultra V Block) and incubated overnight at 4 °C with the primary antibodies against Bcl-2, caspase-3 and HSP70. The sections were then washed and treated with horseradish peroxidase polymer for 15 min at room temperature. 3, 3′-diaminobenzidine (DAKO) was used for visualization of the immunoreaction. Finally, the sections were counterstained with haematoxylin. Negative control was performed by replacing the primary antibody with PBS. The slides were examined and photographed with a photomicroscope attached with a digital camera (Olympus, Tokyo, Japan).

### Statistical analysis

All data are expressed as mean ± SEM. The student’s *t*-test was utilized for statistical comparison between the control and HU groups. A *p*-value of < 0.05 was considered statistically significant.

## Results

### Effect of hindlimb unloading on body weight, gonadosomatic indices and semen parameters

**Fig. 1:**
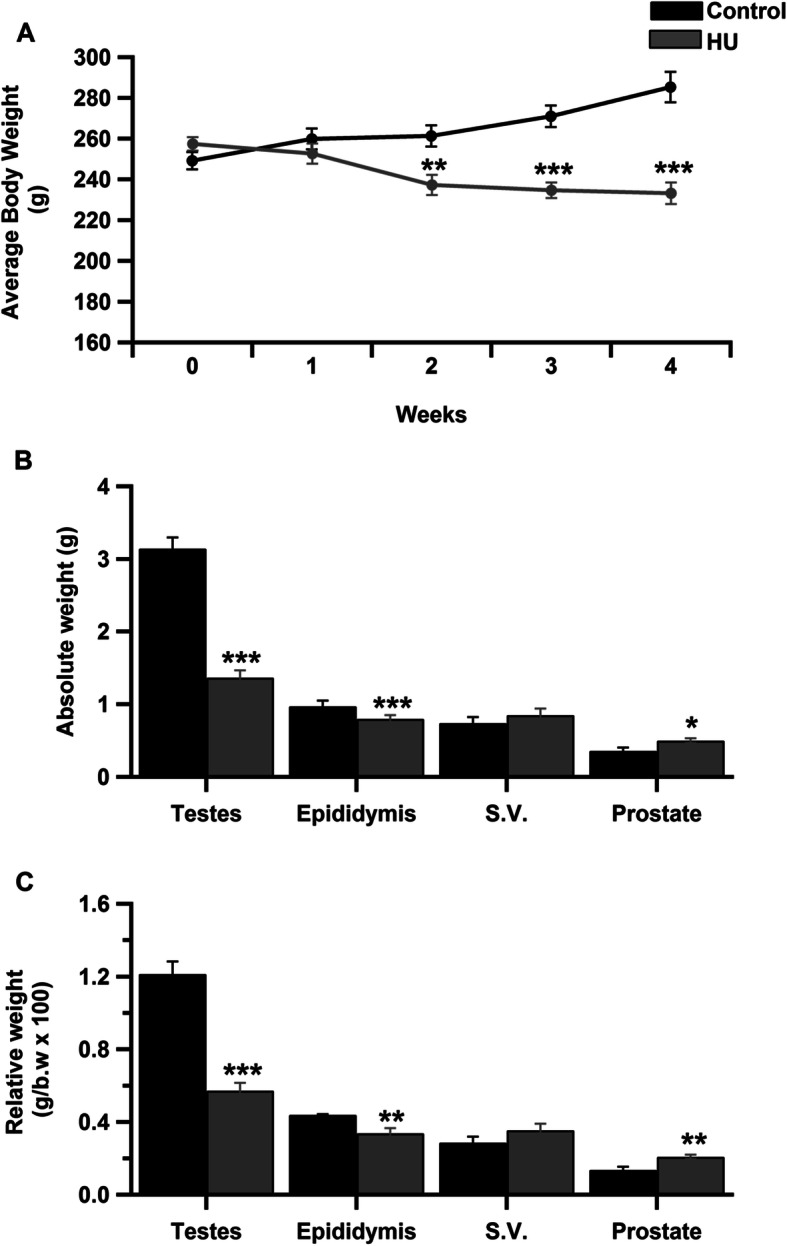
Effect of hindlimb unloading on body weight and gonadosomatic indices in male rats. ***a****:* weakly changes in body weights of the HU group vs control group over the period of the experiment (*n* = 10/group). ***P < 0.001 by Student’s *t*-test. ***b****:* Average weights of testes, epididymides, seminal vesicles and prostate glands in both control and HU group (*n* = 10/group). ***P < 0.001 and *P < 0.05 by Student’s *t*-test respectively. ***c****:* Average somatic indices of testes, epididymides, seminal vesicles and prostate glands in control and HU group (*n* = 10/group). ***P < 0.001 and *P < 0.05 by Student’s *t*-test

Hindlimb unloading for one month significantly decreased body weight (Fig. 1). Body weights of the control rats increased gradually over the 30 days of experiment while the body weights of the HU group began declining sharply after 14 days (Fig. 1a). The average weights of the testes and epididymides significantly decreased in the HU group when compared to the control group (Fig. 1b, *P* < 0.001 by Student’s *t*-test). However, the weights of the prostate glands significantly increased (Fig. 1b, *P* < 0.05 by Student’s *t*-test). The testicular and epididymal somatic indices significantly declined in the HU group (Fig. 1c, *P* < 0.001 and *P* < 0.01 respectively by Student’s *t*-test) while the prostate gland somatic index was significantly elevated in the HU group as compared to the control group (Fig. 1c, *P* < 0.01 by Student’s *t*-test). The average weights and the somatic index of the seminal vesicles showed no significant differences between the control and HU group (Fig. 1b and c). Hindlimb unloading for 30 days induces severe oligospermia (Table. 2, *P* < 0.01 by Student’s *t*-test) with no motile sperms. The sperm abnormalities in this study couldn’t be detected due to oligospermia.

**Table 2:** Effect of hindlimb unloading on semen parameters

Groups	Sperm motility (%)	Sperm count (sperm cell concentration/ml × 125 × 10^**4**^)	Abnormal sperm (%)
**Control**	86.25 ± 2.950	88.125 ± 12.500^a^	23.125 ± 2.065
**Experiment**	No motile sperm	1.125 ± 0.61^b^	–

### Effect of hindlimb unloading on reproductive hormones

**Fig. 2:**
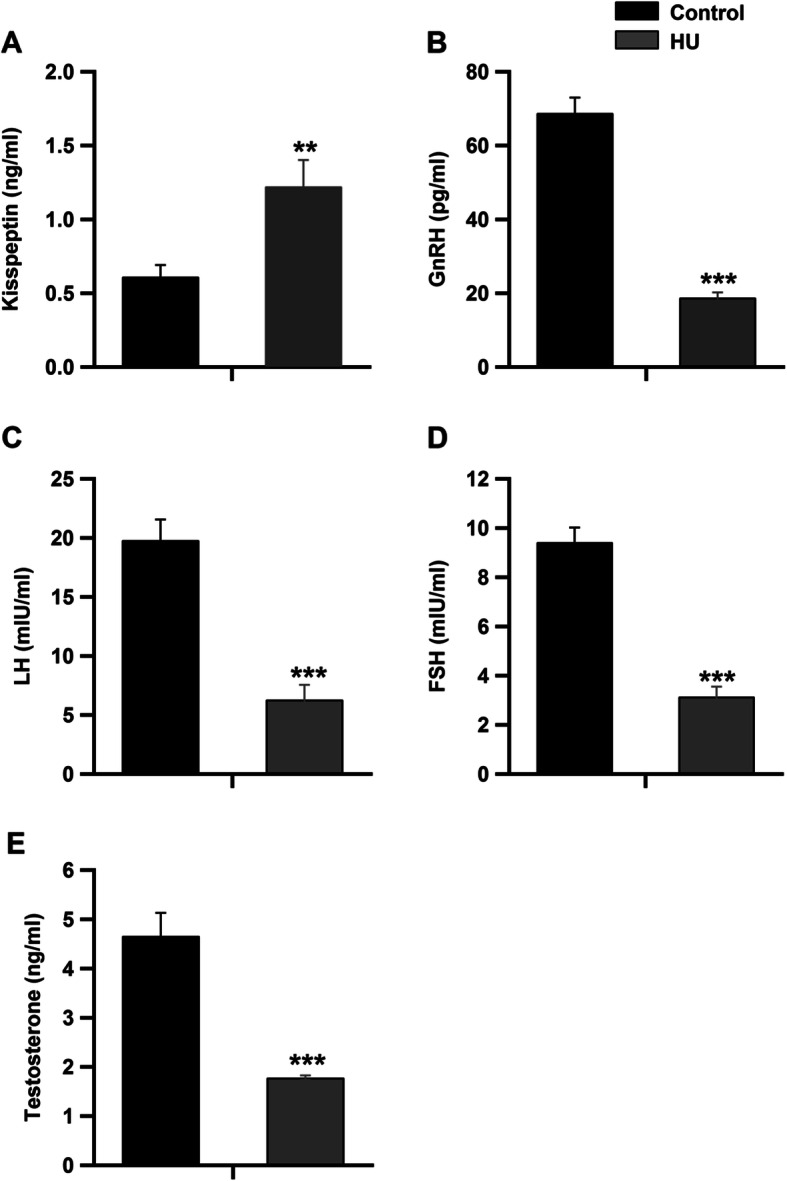
Effect of hindlimb unloading on gonadotropins and sex steroid hormones in male rats. ***a*** and ***b***: Serum levels of kisspeptin (ng/ml) and GnRH (pg/ml) in the control and HU group (*n =* 10/group). ***c*** and ***d***: represent the circulating concentrations of LH and FSH (mIU/ml) in the control and HU group (*n* = 10/group). ***e****:* Show serum concentrations of testosterone hormone (ng/ml) in both control and HU group (*n* = 10/group). **P < 0.01 and ***P < 0.001 by Student’s *t*-test

**Fig. 3:**
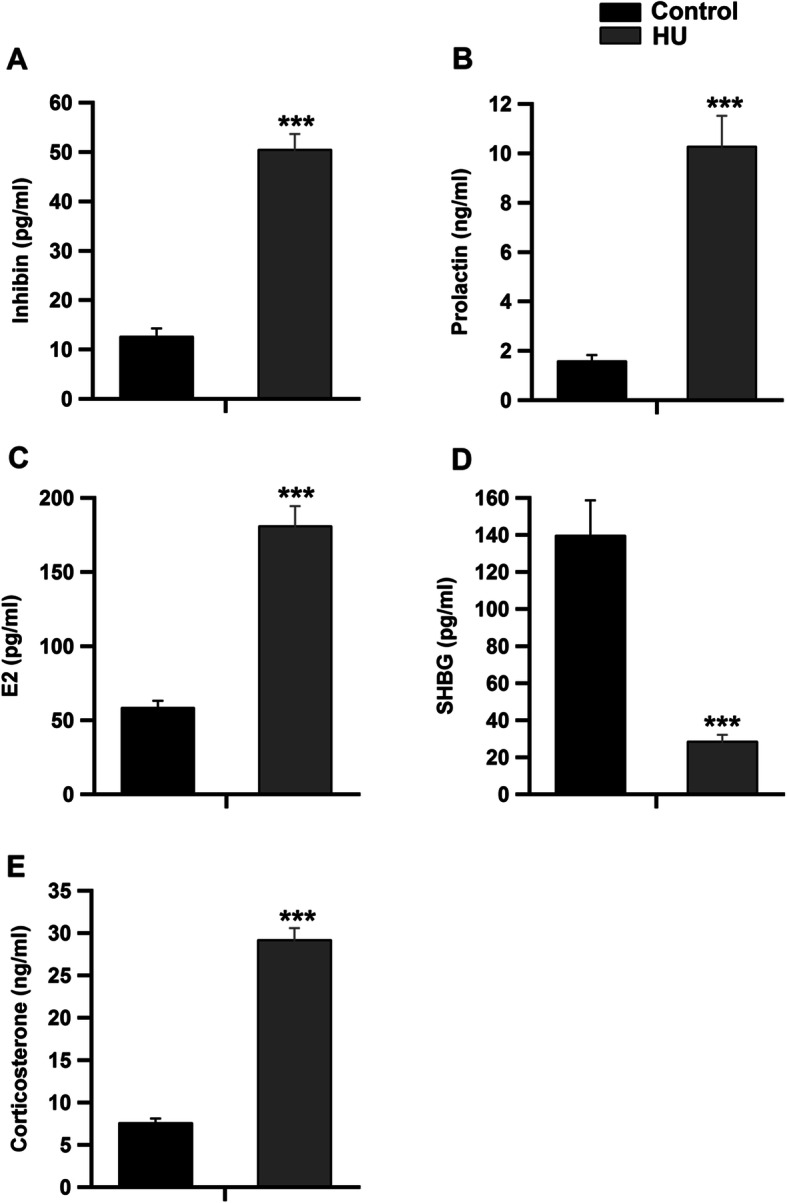
Effect of hindlimb unloading on different reproductive hormones and corticosterone concentrations in male rats. ***a****-****d****:* Circulating concentrations of inhibin, prolactin, E2 and SHBG (pg/ml) in both control and HU group (*n* = 10/group). ***e****:* serum concentrations of corticosterone (ng/ml) in control and HU group (*n* = 10/group). ***P < 0.001 by Student’s *t*-test

**Fig. 4:**
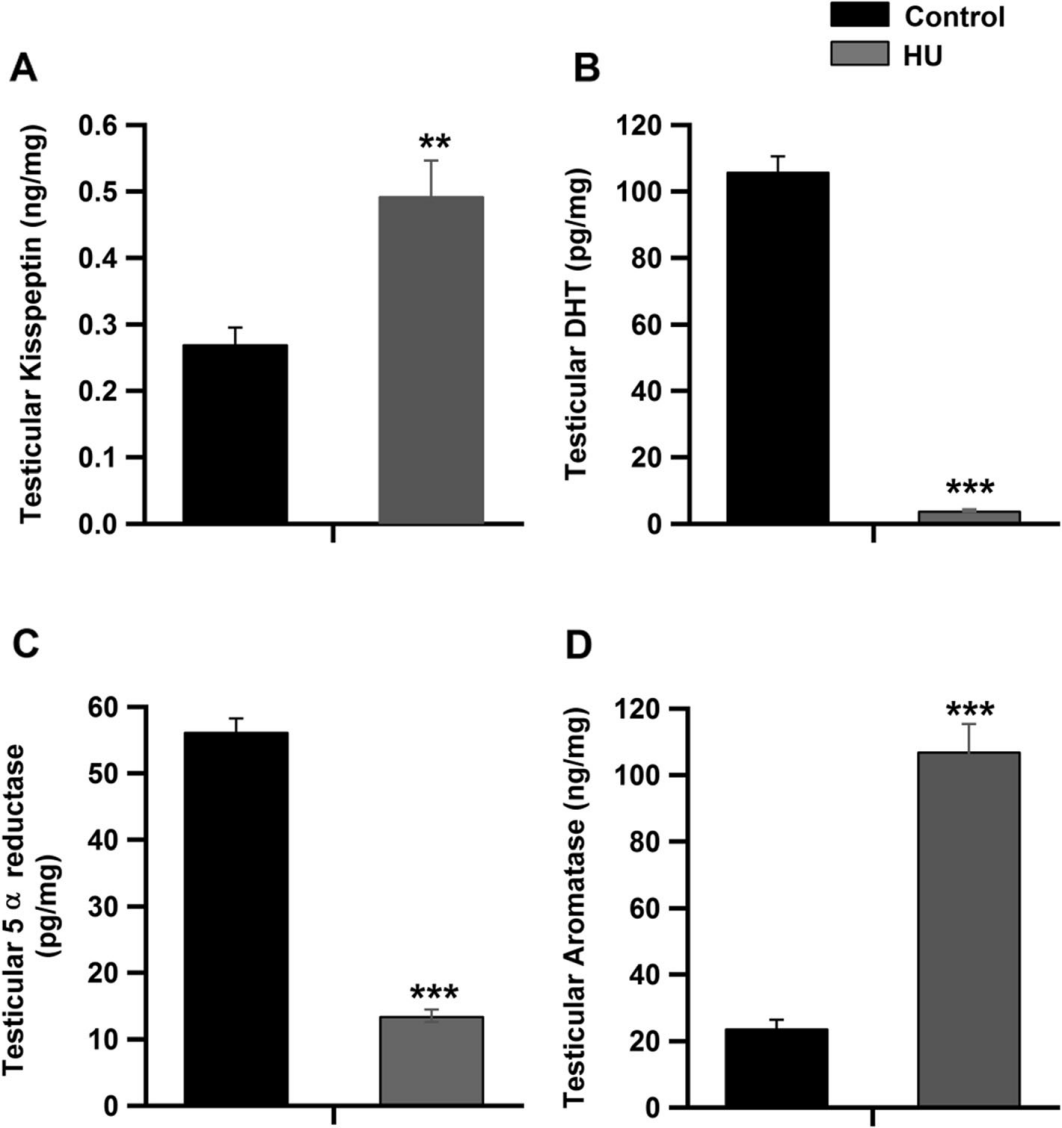
Effect of hindlimb unloading on testicular levels of hormones and enzymes. **a**-**d**: Intratesticular levels of kisspetin (ng/mg), 5α-reductase enzyme (pg/ml) and aromatase (ng/mg) in the control and HU group (n = 10/group). **P < 0.01 and ***P < 0.001 by Student’s t-test.

Serum levels of kisspeptin-1 were significantly increased in the HU group (Fig. 2a, *P* < 0.01 by Student’s *t*-test). However, the concentrations of reproductive hormones, namely GnRH, LH, FSH and testosterone, were significantly inhibited in the HU group compared to the control group (Fig. 2b-e, *P* < 0.001 by Student’s *t*-test). On the contrary, serum concentrations of inhibin, prolactin and E2 hormones were significantly increased in the HU group as compared to the control group (Fig. 3a-c, *P* < 0.001 by Student’s *t*-test). However, the levels of SHBG were significantly reduced in HU group (Fig. 3d, *P* < 0.001 by Student’s *t*-test). Serum concentrations of corticosterone were significantly increased following hindlimb unloading (Fig. 3e, *P* < 0.001 by Student’s *t*-test). The intratesticular levels of kisspeptin-1 were significantly elevated in the HU group as compared to the control group (Fig. 4a, *P* < 0.01 by Student’s *t*-test). However, the intratesticular concentrations of DHT and 5 α-reductase enzyme were suppressed (Fig. 4b and c, *P* < 0.001 by Student’s *t*-test), while the levels of aromatase were increased in the HU group (Fig. 4d, *P* < 0.001 by Student’s *t*-test).

### Effect of hindlimb unloading on HPG axis gene expression

**Fig. 5:**
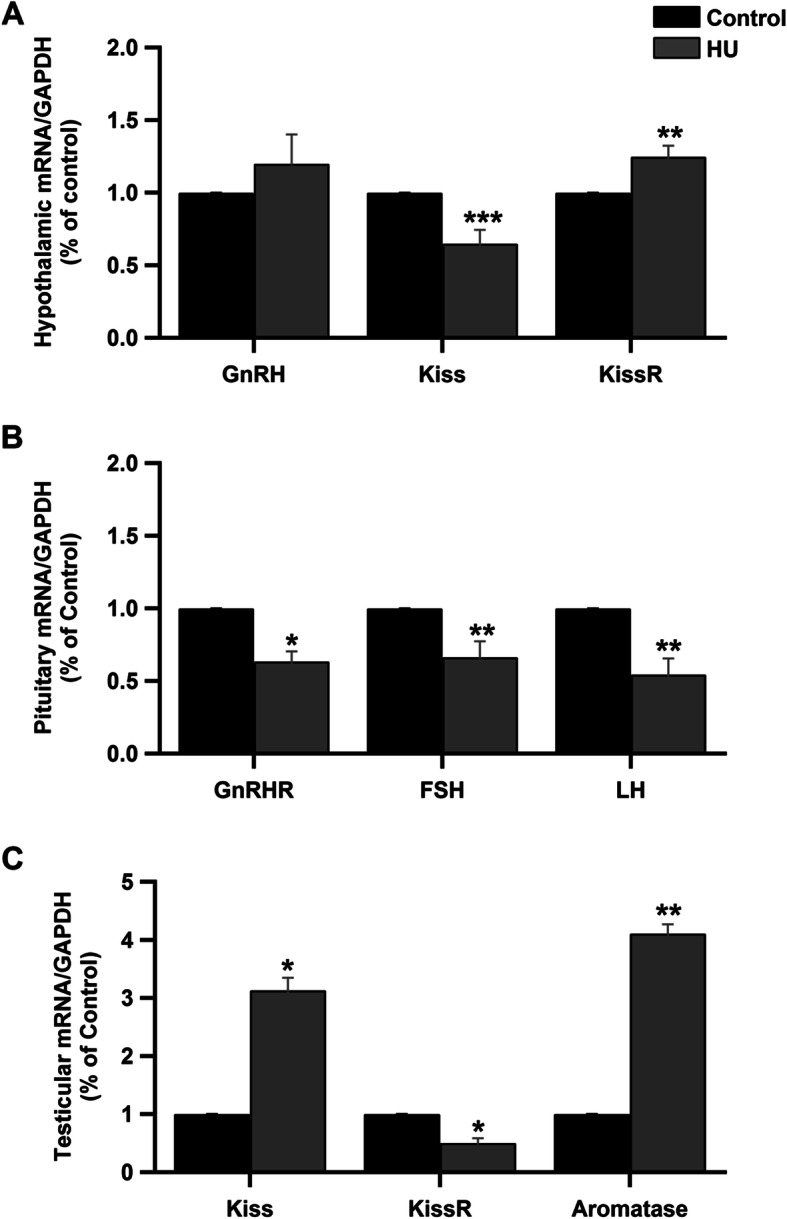
Effect of hindlimb unloading on HPG axis gene expression by RT-PCR. ***a****:* hypothalamic mRNA expression levels of GnRH, Kiss and KissR in control and HU group. ***P < 0.001 and **P < 0.01 by Student’s *t*-test respectively. ***b****:* The mRNA expression levels of GnRHR, FSHβ and LHβ in the pituitary glands of the control and HU group. *P < 0.05 and **P < 0.01 by Student’s *t*-test respectively. ***c****:* Testicular mRNA expression levels of Kiss, KissR and aromatase enzyme in both control and HU group. *P < 0.05 and **P < 0.01 by Student’s *t*-test respectively

The mRNA expression levels of the hypothalamic genes (*GnRH*, *Kiss1* and *Kiss1R*), the pituitary genes (*GnRHR*, *FSHβ* and *LHβ)* and the testicular genes (*Kiss1*, *Kiss1R* and *aromatase*) were investigated. Following hindlimb unloading, the hypothalamic mRNA expression levels of *GnRH* tended to increase but the results were insignificant. However, the expression levels of *Kiss1* mRNA significantly decreased while the mRNA expression levels of *Kiss1R* significantly increased in the HU group as compared to the control group (Fig. 5a, *P* < 0.001 and *P* < 0.01 by Student’s *t*-test respectively). The pituitary gland mRNA expression levels of *GnRHR*, *FSHβ* and *LHβ* were significantly reduced in the HU group (Fig. 5b, *P* < 0.05 and P < 0.01 by Student’s *t*-test respectively). The testicular mRNA expression levels of *Kiss1* gene increased while the expression levels of *Kiss1R* decreased in the HU group (Fig. 5c, *P* < 0.05 by Student’s *t*-test). Furthermore, the expression levels of *aromatase* gene mRNAs were significantly elevated in the HU group as compared to the control group (Fig. 5c, *P* < 0.01 by Student’s *t*-test).

### Effect of hindlimb unloading on antioxidant enzymes, lipid peroxidation and NO

**Fig. 6:**
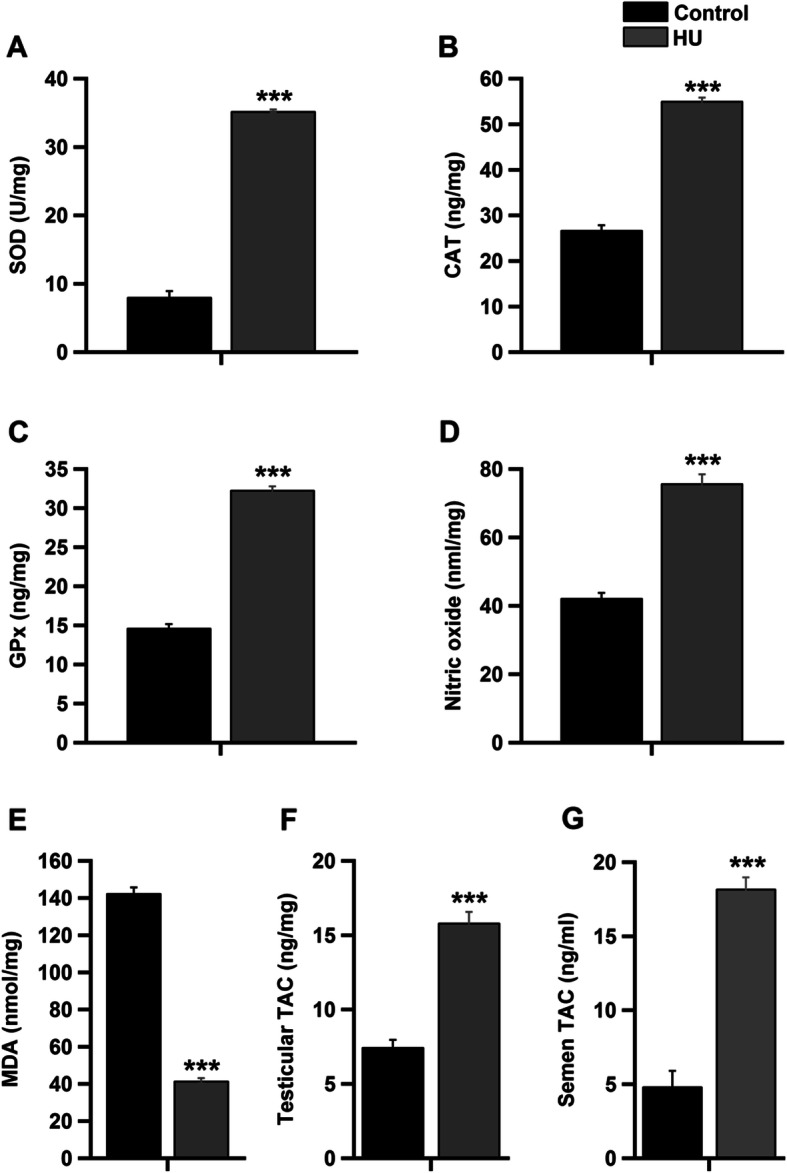
Effect of hindlimb unloading on antioxidant enzymes, lipid peroxidation, NO and TAC. Testicular levels of ***a****:* SOD (U/mg), ***b****:* CAT (ng/mg), ***c****:* GPx (ng/mg), ***d****:* NO (nml/mg) and ***e****:* MDA (nmol/mg) in the control and HU group (*n* = 10/group). ***f*** and ***g***: testicular and seminal levels of TAC (ng/mg) in the control and HU group respectively (*n* = 10/group). ***P < 0.001 by Student’s *t*-test

The testicular antioxidant enzymes SOD, CAT and GPx, in addition to NO, significantly increased in the HU group (Fig. 6a-d, *P* < 0.001 by Student’s *t*-test). Conversely, the testicular levels of the lipid peroxidation indicator MDA significantly declined in the HU group as compared to the control group (Fig. 6e, *P* < 0.001 by Student’s *t*-test). Both testicular and seminal TAC were significantly elevated in HU group (Fig. 6f and g, *P* < 0.001 by Student’s *t*-test).

### Effect of hindlimb unloading on the immunohistochemical expression of Bcl-2, Caspase-3 and HSP-70

**Fig. 7:**
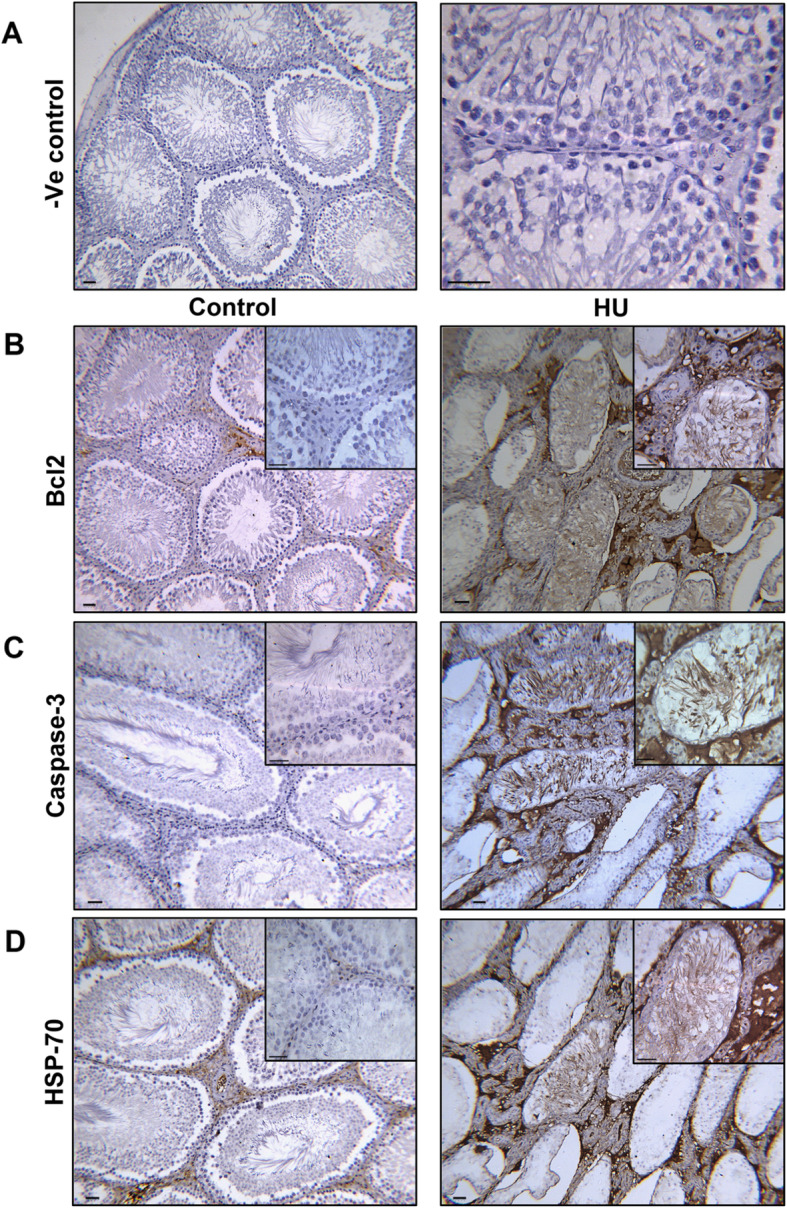
Effect of hindlimb unloading on apoptotic markers and HSP-70 immunohistochemistry. ***a****:* a negative control slide. ***b****:* Bcl-2 immunoreaction in the testes of the control and HU group. ***c****:* Testicular expression of caspase-3 in the control and HU group. ***d****:* The immunohistochemical expression of HSP-70 in the testes of the control and HU group. Scale bar = 100 μM

Both Bcl-2 and caspase-3 showed no expression in the testes of the control group; however, the expression markedly increased in the testes of the HU group, especially within seminiferous tubules, interstitial cells and spermatozoa (Fig. 7b and c). In the testes of the control group, the expression of HSP-70 was faint and the localization was restricted to the interstitial cells, but the expression was distinctly increased in the seminiferous tubules, spermatozoa and interstitial cells of the HU group (Fig. 7d).

### Effect of hindlimb unloading on reproductive organs morphology

**Fig. 8:**
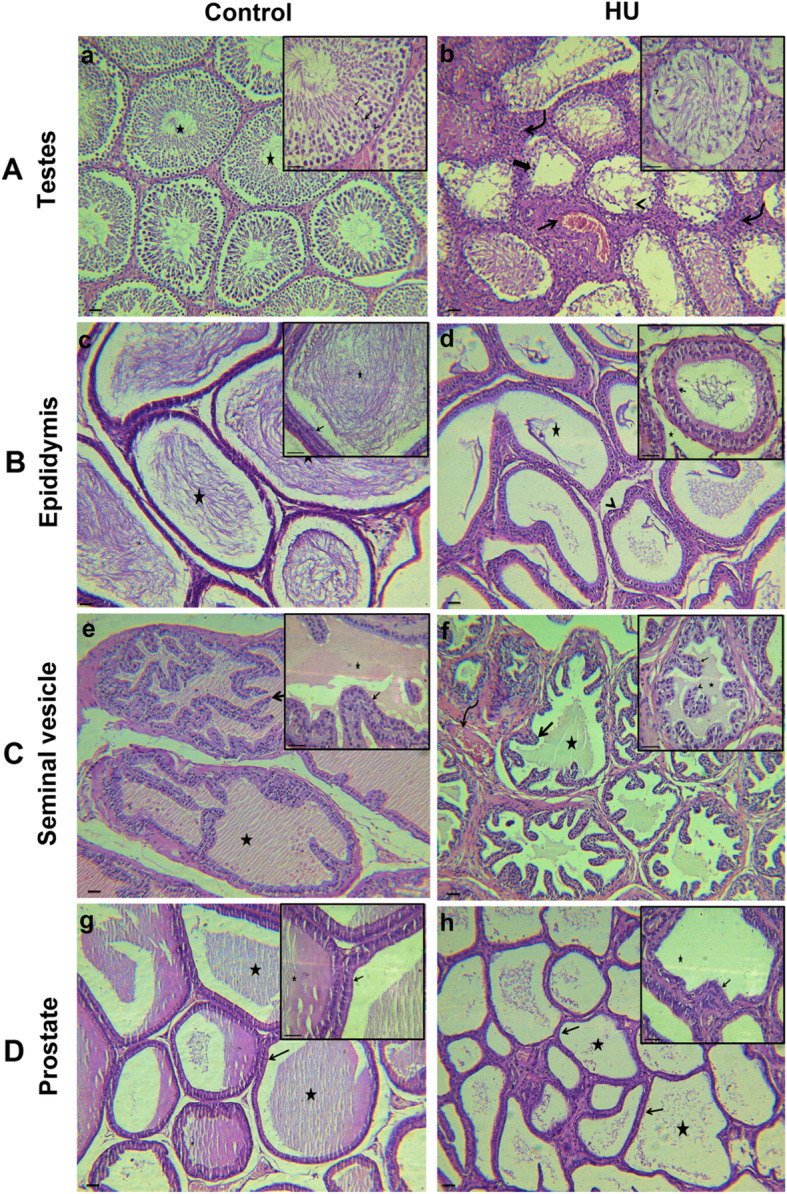
Effect of hindlimb unloading on reproductive organs morphology. ***a****:* photomicrograph of testes of the control group showing normal spermatogonia **(arrow head)** with preserved spermatogenesis **(arrow)** and spermiogenesis **(curved arrow)** with the presence of normal population of sperms within seminiferous tubules lumina **(stars)**. The testes of the HU group demonstrating necrotic spermatogenic cells **(arrow heads)**, atrophied seminiferous tubules **(thick arrow)** with increase in the interstitial tissues **(curved arrows)** and mild congestion of the testicular blood vessels (**arrow).** ***b****:* Photomicrograph of the epididymis of the control group revealing normal columnar epithelial lining the epididymis (**arrow**) with large number of mature sperms inside the tubules **(stars)**. The epididymis of the HU group showing reduction of the collected sperms **(stars)**, intertubular edema (**arrow heads**) and relatively normal ciliated pseudostratified columnar epithelium lining the epididymal tubules (**arrow**). ***c****:* Seminal vesicle of the control group showing columnar epithelial lining the secretory acini **(arrow)** with normal eosinophilic secretion **(stars)**. The seminal vesicles of the HU group showing mild interstitial congestion **(curved arrow)**, mild hyperplastic epithelial lining **(arrow)** with secretory products **(star)** and desquamated epithelium on their lumina **(arrow head)**. ***d****:* Photomicrograph of the control group prostate showing normal glandular tissue with columnar epithelial lining **(arrows)** and acidophilic secretion **(stars)**. The prostate gland of HU group exhibiting cuboidal epithelial lining the acini **(arrows)** with less secretory products **(stars)**. Scale bar = 100 μM

Histological examination of the testes from the control groups revealed normal morphological patterns of the seminiferous tubules with preserved spermatogonia, spermatocytes, spermatids and sperms. Moreover, a normal population and structures of both leydig cells and sertoli cells were also observed. On the contrary, the testes of the HU groups showed testicular lesions, representing the destruction of the seminiferous tubules with the disappearance of a large number of spermatogonia and spermatocytes. Some seminiferous tubules lacked any spermatozoa and showed atrophic and necrotic changes. Moreover, characteristic increases in the interstitial tissues occurred. Mild congestion of the testicular blood vessel was also observed (Fig. 8a).

The examined sections from the epididymis of the control groups demonstrated normal cuboidal to columnar lining epithelium of the tubules with long microvilli. The epididymal lumina filled with stored spermatozoa. However, the epididymal tubules of the HU groups displayed a severe reduction in the amount of stored sperm with edematous interstitial tissue (Fig. 8b).

The seminal vesicles of the control groups showed normal secretory acini with columnar epithelial lining and papillary projections, as well as normal eosinophilic secretion. On the contrary, the seminal vesicles of the HU groups revealed mild interstitial congestion. The epithelial lining the glandular tissue showed mild hyperplastic changes with secretory products and desquamated epithelium in their lumina (Fig. 8c).

Sections from the prostates of the control groups showed apparently normal glandular tissue with columnar epithelial lining and acidophilic secretion within the alveolar lumina. However, sections from the prostate glands of the HU groups demonstrated less active glandular tissue with cuboidal epithelial lining the acini and less secretory products (Fig. 8d).

## Discussion

With the increased investment in space flights and the ability of humans to live outside the earth’s atmosphere, it is crucial to assess the impact of space-associated problems like weightlessness and altered gravity on human bodies, particularly the reproductive system. The hindlimb unloading model has been widely used to simulate the microgravity environment. The hypothalamic-pituitary-testicular (HPT) axis is the key regulator of male reproductive functions and normal spermatogenesis [16]. The hypothalamic GnRH binds with the GnRHR in the anterior pituitary, which further stimulates the secretion of LH and FSH; LH promotes testosterone secretion from the leydig cells, which further augments sperm production, while FSH stimulates sertoli cells and assists the process of spermatogenesis [17].

The results of this study revealed that hindlimb unloading for 30 days suppresses the HPT axis in adult male rats, via a decrease in the circulating levels of gonadotropins resulting from reduced mRNA expression levels of *FSHβ* and *LHβ* in the pituitary gland and the subsequent decrease in serum testosterone concentrations. The decreased serum levels of FSH may be attributed to the inhibitory effect of inhibin, which was significantly elevated following hindlimb unloading. Previous research has reported that testosterone can be metabolized by 5*α*-reductase to the more potent androgen DHT, which plays a fundamental role in supporting spermatogenesis in adult rats [18]. In this study, the testicular level of DHT was significantly inhibited by hindlimb unloading via decreased levels of intratesticular 5*α*-reductase (Fig. 4). Alternatively, testosterone can be also metabolized to E2 by aromatization [19].

Hindlimb unloading for 30 days markedly upregulated the mRNA expression levels of *aromatase* as well as the intratesticular concentrations of aromatase and hence, the serum levels of E2 were elevated. Previous studies have demonstrated the detrimental effects of E2 on male fertility [20]. High doses of E2 have been shown to inhibit reproductive behavior, induce azoospermia and abolish fertility in adult rats [21]. Moreover, excessive intratesticular E2 concentrations decreased intratesticular androgens and induced spermiation failure in E2-treated rats [22, 23]. Furthermore, in male humans, E2 hinders gonadotropin release through the hypothalamus and pituitary gland [24]. Enhancement of prolactin secretion has been reported by E2 direct action at the pituitary level [25]. This study revealed elevated levels of circulating prolactin in the HU group. Although the functional significance of prolactin in male reproduction is unclear, the hormone has been linked mainly with male infertility. Acute hyperprolactinemia suppresses testosterone synthesis and male fertility by inhibiting the secretion of GnRH [26], while a moderate increase in circulating prolactin has been shown to suppress both LH and FSH but not testosterone [21]. Therefore, high circulating levels of prolactin may contibute to hindlimb unloading-induced hypogonadism via inhibition of gonadotropins and testosterone synthesis. The bioavailability of sex steroids and their physiological responses are regulated by SHBG [27]. Since prolactin has been demonstrated as an inhibitor of SHBG [28], hyperprolactinemia observed in the present study may contribute to the reduced plasma levels of SHBG in HU group.

Increasing evidence has shown that the *Kiss1* gene and its product kisspeptin play a fundamental role in regulating the HPG axis, the hypothalamic GnRH secretion and thereby gonadotropins secretion [29]. *Kiss1* and its receptors *Kiss1R* are not only expressed in the hypothalamus, but also in human and rodent testes [30, 31]. Previous research has demonstrated that the mRNA expression levels of *Kiss1* are 17 times higher in the testes than in the hypothalamus [32], indicating that the Kiss1/Kiss1R may manage important testicular functions. In this study, hindlimb unloading downregulated the hypothalamic mRNA expression levels of *Kiss1* while upregulating the mRNA expression levels of *Kiss1R*. However, the expression of *Kiss1* mRNA at the testicular level was significantly upregulated whereas the *Kiss1R* mRNA was downregulated, indicating that at most one element of kiss1-Kiss1R signaling is downregulated in response to hindlimb unloading.

A spaceflight-induced increase in corticosterone concentrations has also been reported [33]. Furthermore, the attainment of corticosterone concentrations similar to those observed in acute [34] or chronic [35, 36] stressors has been shown to downregulate *Kiss1* and upregulate *Kiss1R* mRNA expression in the hypothalamic medial preoptic area and arcuate nucleus. Moreover, previous research has reported reduced expression levels of *Kiss1* mRNA in the rat arcuate nucleus by psychological, restraint and immunological stressors [37]. As well, fasting has been demonstrated to induce a similar decline in the expression of *Kiss1* and elevation in *Kiss1R* in the whole hypothalamus in rats [38] and mice [39], suggesting that an increase in the expression of *Kiss1R* may attempt to compensate for the decrease in *Kiss1* gene expression by producing a sensitivity to the effects of kisspeptin [37]. This study indicated that the hindlimb unloading-induced inhibitory effect on the hypothalamic *Kiss1* mRNA expression may therefore be in part due to the high circulating levels of corticosterone and/or the high testicular levels of kisspeptin and *Kiss1* mRNAs, which may convey a negative feedback signal to control the hypothalamic *Kiss1* production and the reproductive functions.

Oxidative stress may threaten animal health, particularly in the reproductive system. Sperm fertility largely relies on the presence of antioxidant systems, and both spermatogenesis [40] and Leydig cell steroidogenesis [41] are susceptible to oxidative stress. In addition, lipid peroxidation factors in testicular dysfunction [40]*.* A spaceflight-induced increase in lipid peroxidation has been reported in rodents [42]*.* Moreover, hindlimb unloading has been shown to increase oxidative stress markers in rat’s liver, pancreas, kidneys, intestine, lung, heart, and brain [43]. Additionally, research has demonstrated a decrease in the SOD concentrations and an increase in the reactive oxygen species (ROS) generation by eliciting microgravity in the rat’s hippocampus [44]. However, the results of this study revealed increases in the testicular levels of SOD, CAT, GPx and TAC, as well as seminal TAC, following hindlimb unloading. Notably, MDA concentrations were suppressed in the HU group (Fig. 6). As mammalian testes are particularly sensitive to oxidative stress [45], the increased activities of the antioxidant enzymes observed in the present study more likely represent an adaptive mechanism through which the organisms protect themselves from the deleterious effects of ROS.

The cytotoxic effects of ROS can be ameliorated or potentiated by NO [46, 47]. Moreover, the biological function of NO as a destructive or a protective agent is determined by the balance between NO and ROS [47], the tissue, source, and environment [48]. A correlation between increased ROS production, a decrease in testosterone [49] and an elevation in NO [50] were reported. Stress-induced increases in NO also reduce testosterone production [51, 52]. Moreover, steroidogenesis by the Leydig cells and the adrenal cortex has been shown to be suppressed by NO [51, 53]. In this study, hindlimb unloading significantly elevated the testicular concentrations of NO (Fig. 6d). Notable increases in the activity of nitric oxide synthases and NO production were also demonstrated in rats subjected to tail suspension for 14 days [54]. NO plays a fundamental role in sperm production and motility. Low concentrations of NO enhance sperm motility [55], whereas high concentrations abolish it [56]; an adverse correlation between NO and sperm motility, morphology and concentration has been reported [57]. Consequently, the decreased testosterone concentrations and subsequent decrease in sperm concentrations and suppressed motility demonstrated following hindlimb unloading may be in part attributed to the elevated concentrations of intratesticular NO.

Cellular protection against ROS includes not only antioxidant enzymes but also expression of heat shock proteins (HSP). Mammalian cells are rich in HSP70 that inhibits oxidative stress-induced apoptosis [58]. HSP70 normally presents in unstressed cells at a basal level and is strongly expressed following stress [59]. HSP70 engages in cellular division during spermatogenesis and produces steroid hormones in leydig cells [60]. Previous studies have demonstrated that Bcl-2 serves as a potent anti-apoptotic protein [61] while caspase-3 has been reported as a pro-apoptotic protein [62]. The present results showed an overall increase in caspase-3, Bcl-2 and HSP70 immunostaining in the seminiferous tubules, spermatozoa and testicular interstitial cells of the HU group. LH, FSH and testosterone deprivation induce apoptosis in rat testes and block spermatogenesis [63–65]. Moreover, caspase-3 activation mediates germ cell apoptosis as a result of decreased intratesticular testosterone concentration [62]. Therefore, this study proposed that hindlimb unloading-induced decreases in the concentrations of LH, FSH and testosterone may prompt testicular apoptosis as evidenced by the morphological study (Fig. 8) via induction of caspase-3, which further upregulates HSP70 and Bcl-2 as a protective mechanism to inhibit the apoptotic cascades downstream caspase-3 activation.

## Conclusions

**Fig. 9:**
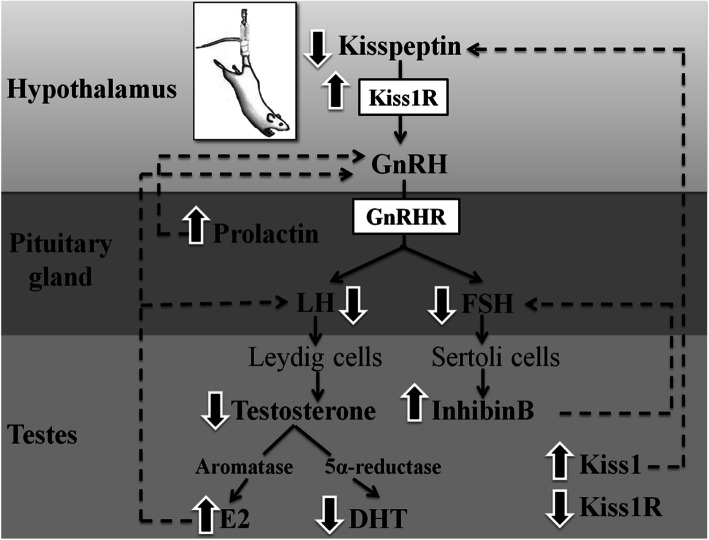
Proposed scheme of the potential effects of hindlimb unloading on HPG axis in male rats. Solid lines indicate stimulation while dashed lines indicate inhibition

In summary, hindlimb unloading suppresses the reproductive functions in male rats via various mechanisms including 1) inhibition of the kisspeptin-HPG axis and subsequent downregulation of mRNAs expression levels of *FSHβ* and *LHβ*, with a decrease in gonadotropin secretion that inhibits both spermatogenesis and steroidogenesis; 2) Suppressive effects of hyperprolactinemia on gonadotropin and testosterone synthesis with a resulting hypogonadism; 3) An inhibitory effect of high inhibin concentrations on FSH production; and 4) Upregulation of testicular aromatase mRNA expression levels and thereby an elevation in E2 concentrations that suppress gonadotropin release (Fig. 9). Furthermore, hindlimb unloading represents an environmental stress in which organisms try to adapt themselves via an enhancement of antioxidant enzymes activities and an improvement of anti-apoptotic factors as HSP70 and Bcl2. Finally, some precautions should be considered in order to protect space travelers from the adverse effects of microgravity on their reproductive system.

## Data Availability

The datasets used and/or analyzed during the current study are available from the corresponding author on reasonable request.

## Abbreviations

CAT: Catalase
DHT: Dihydrotestosterone
E2: Estradiol
FSH: Follicle stimulating hormone
GPx: Glutathione peroxidase
GnRH: Gonadotropin releasing hormone
HSP70: Heat shock protein 70
Kiss-1: Kisspeptin-1
Kiss-1R: Kisspeptin-1 receptors
LH: Luteinizing hormone
MDA: Malondialdehyde
NO: Nitric oxide
ROS: Reactive oxygen species
SHBG: Sex hormone binding globulin
SOD: Superoxide dismutase
TAC: Total antioxidant capacity
